# Supplementary material for: Osteology of the axial skeleton of Aucasaurus garridoi: phylogenetic and paleobiological inferences
Source: PeerJ. 2023 Nov 14;11:e16236. doi: 10.7717/peerj.16236 (PMC10655716; doi:10.7717/peerj.16236)
Supplement: Supplemental Information 1 — Notation: AnCH, anterior centrum height; AnCW, anterior centrum width; CENL, centrum length; IZL, interzygapophyseal length between anterior rim of right prezygapophysis to posterior rim of right postzygapophysis; IZW, interzygapophyseal width between lateral rim of postzygapophyses; MidW, midcentral width; NSH, neural spine height; NSL, neural spine length; NSW, neural spine width; PoCH, posterior centrum height; PoCW, posterior centrum width; ToVH, total vertebral height; TP/CT, ratio between the transversal length of the transverse process and the centrum anteroposterior centrum length; *, incomplete measurement due to missing bone; -, measurement not applicable. [file peerj-11-16236-s001.docx]

**Table S1.** Principal measurements in cm of vertebrae of Aucasaurus garridoi MCF-PVPH-236.

| VERTEBRA | CENL | AnCW | AnCH | PoCW | PoCH | MidW | ToVH |
| --- | --- | --- | --- | --- | --- | --- | --- |
| Atlas | 2,3 | 4,5 | 4,2 | 6 | 4,8 | 5,5 | 6,4* |
| Dorsal 2 | 11* | - | - | 5,5* | 6,1* | - | - |
| Dorsal 3 | 7,7 | 5,1* | 7,5 | 4,9* | 7,5 | 2,4* | 21,6 |
| Dorsal 4 | 8,6 | 5,7* | 9,2 | 6,5* | 8,6 | 2,2* | 19,5 |
| Dorsal 5 | 8,7 | 6,3* | 9,2 | 6* | 8,6 | 1,2* | 21,9 |
| Dorsal 6 | 9,2 | 6,2 | 9,6 | 6,2* | 8,5* | 2,3* | 23,5 |
| Dorsal 7 | - | - | - | - | - | - | - |
| Dorsal post. | 7,8 | 9,1 | 8* | 8,9* | 9,2* | 4 | - |
| Dorsal post. | 9 | 6,2* | - | 7* | 8,3 | 4,5 | 20,5* |
| Sacrum | 33,4* | - | - | - | - | - | - |
| Sacral 1 | 10,3 | 5,5* | - | 2,7 | - | 2,5 | - |
| Sacral 2 | 4,7 | 2,6 | - | - | - | - | - |
| Sacral 3 | - | - | - | - | - | - | - |
| Sacral 4 | - | - | - | - | - | - | 25,5 |
| Sacral 5 | 9,3 | 2,7 | 5,2* | 4,3 | 7,4 | 3,6 | 26,6 |
| Sacral 6 | 9,1 | 4,3 | 5,2* | 9,5 | 10,4 | - | 28,2 |
| Caudal 1 | 10 | 9,9 | 9 | 8,4* | 9,3 | 5,6 | 27,9 |
| Caudal 2 | 10,5 | 8,8 | 10 | 8 | 8,4 | 4,2 | 24,8* |
| Caudal 3 | 10,1 | 7,9 | 9 | 7,4 | 7,8 | 4,1 | 23,3* |
| Caudal 4 | 9,4 | 7,2 | 8 | 6,9 | 7,5 | 3,7 | 24,2* |
| Caudal 5 | 6,5 | 7,4 | 7,3 | 3,8 | 6,8 | 3,7 | 23,4 |
| Caudal 6 | 6,8 | 3,8 | 6,8 | 6 | 6,5 | 2,8 | 21,7 |
| Caudal 7 | 9,1 | 5,8 | 6,3 | 5,3 | 5,8 | 2,8 | 21,3 |
| Caudal 8 | 9 | 5,4 | 6,7 | 5,4 | 6,3 | 2,6* | 19,4 |
| Caudal 9 | 9,2 | 5,9 | 6,2 | 5,5 | 5,9 | 3,2 | - |
| Caudal 10 | 8,8 | 5,6 | 5,9 | 5,8 | 5,8 | 3,2 | - |
| Caudal 11 | 8,7 | 5,6 | 5,7 | 5,6 | 5,6 | 3 | - |
| Caudal 12 | 8,3 | 5,4 | 5,3 | 5,4 | 5,6 | 3 | - |
| Caudal 13 | 6,1* | 5,4 | 5,2 | - | - | 3 | - |

| VERTEBRA | NSH | NSL | NSW | IZW | IZL | TP/CT |
| --- | --- | --- | --- | --- | --- | --- |
| Atlas | - | - | - | - | - | - |
| Dorsal 2 | 8,2* | 3,2* | 2,2* | - | - | - |
| Dorsal 3 | 7,1* | 4,6 | 2,4* | - | 9,2* | - |
| Dorsal 4 | 7,9* | 3,7* | 3* | - | 9,4 | - |
| Dorsal 5 | 8,5 | 5,6 | 3,1 | - | - | - |
| Dorsal 6 | 9,6 | 7,1 | 3,1 | - | - | - |
| Dorsal 7 | 10,1* | 7,7 | 3,5 | - | - | - |
| Dorsal post. | - | - | - | - | - | - |
| Dorsal post. | 11,4* | - | - | - | - | - |
| Sacrum | 50* | - | - | - | - | - |
| Sacral 1 | - | - | - | - | - | - |
| Sacral 2 | - | - | - | - | - | - |
| Sacral 3 | - | - | - | - | - | - |
| Sacral 4 | - | 9,6* | - | - | - | - |
| Sacral 5 | - | 8,9 | - | - | - | - |
| Sacral 6 | - | 10,8 | - | - | - | - |
| Caudal 1 | 10,5 | 5,5 | 1,9 | 3 | 10,6* | 1,3 |
| Caudal 2 | 5,6* | 5,7 | 1,8 | 2,4* | 10,9* | 1,12 |
| Caudal 3 | 5,2* | 6,1 | 1,7 | 2,3* | 10,8* | 1,23 |
| Caudal 4 | 9,3* | 6,1 | 1,3 | 2,4* | 8,6 | 1,41 |
| Caudal 5 | 8,9 | 6 | 1,3 | - | 7,3* | 2,04 |
| Caudal 6 | 8,7 | 4,3 | 1,1 | 2,7* | 7,3* | 2,01 |
| Caudal 7 | 9,2 | 5,7 | 1,6 | - | 9,3* | 1,17 |
| Caudal 8 | 8,5 | 5,3 | 1,5 | - | 9,4* | 1,32 |
| Caudal 9 | - | 4,7 | 1,5 | - | 10,8* | 1,34 |
| Caudal 10 | - | - | 1,4 | - | 8,5* | 1,42 |
| Caudal 11 | - | 3,7 | 1,4 | - | 9,7* | 1,43 |
| Caudal 12 | - | 3,5 | 1,4 | - | 9,9 | 1,28 |
| Caudal 13 | - | 3,1 | 1,4 | - | 8,4 | - |

Notation: **AnCH**, anterior centrum height; **AnCW**, anterior centrum width; **CENL**, centrum length; **IZL**, interzygapophyseal length between anterior rim of right prezygapophysis to posterior rim of right postzygapophysis; **IZW**, interzygapophyseal width between lateral rim of postzygapophyses; **MidW**, midcentral width; **NSH**, neural spine height; **NSL**, neural spine length; **NSW**, neural spine width; **PoCH**, posterior centrum height; **PoCW**, posterior centrum width; **ToVH**, total vertebral height; **TP/CT**, ratio between the transversal length of the transverse process and the centrum anteroposterior centrum length; *, incomplete measurement due to missing bone; -, measurement not applicable.
