# Supplementary material for: Osteology of the axial skeleton of Aucasaurus garridoi: phylogenetic and paleobiological inferences
Source: PeerJ. 2023 Nov 14;11:e16236. doi: 10.7717/peerj.16236 (PMC10655716; doi:10.7717/peerj.16236)
Supplement: Supplemental Information 2 — Notation: L, proximodistal length; *, incomplete measurement due to missing bone. [file peerj-11-16236-s002.docx]

**Table S2.** Principal measurements in cm of haemal arches of *Aucasaurus garridoi* MCF-PVPH-236.

| ELEMENT | L |
| --- | --- |
| Haemal arch 1 | 24,7 |
| Haemal arch 2 | 26,2 |
| Haemal arch 3 | 22,4 |
| Haemal arch 4 | 25,5 |
| Haemal arch 5 | 23,9 |
| Haemal arch 6 | 17,7 |
| Haemal arch 7 | 26,3 |
| Haemal arch 8 | 24,1 |
| Haemal arch 9 | 22,9 |
| Haemal arch 10 | 19,2 |
| Haemal arch 11 | 16,2* |
| Haemal arch 12 | 19,9 |
| Haemal arch 13 | 12,8* |

Notation: **L**, proximodistal length; *, incomplete measurement due to missing bone.
