# Supplementary material for: Osteology of the axial skeleton of Aucasaurus garridoi: phylogenetic and paleobiological inferences
Source: PeerJ. 2023 Nov 14;11:e16236. doi: 10.7717/peerj.16236 (PMC10655716; doi:10.7717/peerj.16236)
Supplement: Supplemental Information 3 — Notation: pocdf, postzygapophyseal centrodiapophyseal fossa; *, unable to measure due to rupture or missing bone; -, measurement not applicable. [file peerj-11-16236-s003.docx]

**Table S3.** Principal measurements in cm of foramina present in caudal vertebrae of Aucasaurus garridoi MCF-PVPH-236.

| VERTEBRA | NEURAL SPINE  FORAMINA |  | POCDF FORAMINA |  |
| --- | --- | --- | --- | --- |
|  | RIGHT | LEFT | RIGHT | LEFT |
| CAUDAL 1 | - | - | 0,4 length  0,3 width | 0,5 length  0,2 width |
| CAUDAL 2 | - | - | 0,6 length  0,4 width | 0,7 length  0,4 width |
| CAUDAL 3 | - | - | 0,9 length  0,5 width | 1 length  0,3 width |
| CAUDAL 4 | - | - | 0,4 length  0,1 width | 0,5 length  0,3 width |
| CAUDAL 5 | Missed data | 1 length  0,5 width | Missed data | Missed data |
| CAUDAL 6 | 1,2 length  0,5 width | 1 length  0,5 width | Missed data | 1 length  0,5 width |
| CAUDAL 7 | 1,1 length  0,9 width | Missed data | 0,5 length  0,4 width | 1,1 length  0,5 width |
| CAUDAL 8 | 1,1 length  1 width | 1,1 length  0,3 width | 0,9 length  0,2 width | 0,8 length  0,2 width |
| CAUDAL 9 | 1 length  0,4 width | 1 length  0,4 width | 0,2 length  0,2 width | * |
| CAUDAL 10 | 1 length  0,4 width | 1,1 length  0,4 width | - | - |
| CAUDAL 11 | 0,6 length  0,4 width | 1,5* length  0,4 width | - | - |
| CAUDAL 12 | Shallow depression | Shallow depression | * | * |
| CAUDAL 13 | Shallow depression | Shallow depression | - | - |

Notation: pocdf, postzygapophyseal centrodiapophyseal fossa; *, unable to measure due to rupture or missing bone; -, measurement not applicable.
