# Supplementary material for: Osteology of the axial skeleton of Aucasaurus garridoi: phylogenetic and paleobiological inferences
Source: PeerJ. 2023 Nov 14;11:e16236. doi: 10.7717/peerj.16236 (PMC10655716; doi:10.7717/peerj.16236)
Supplement: Supplemental Information 4 [file peerj-11-16236-s004.docx]

**Character List**

1. External surface of maxilla and nasal: smooth (0); sculptured (1) (Carrano & Sampson, 2008).

2. External surface of postorbital, lacrimal, and jugal: smooth (0); sculptured (1) (Carrano & Sampson, 2008).

3. Premaxilla, maxillary process: well developed (0); reduced to a short triangle or absent (1) (modified from Carrano & Sampson, 2008).

4. Premaxilla, subnarial foramen: enclosed (0); reduced/open dorsally (1) (Carrano & Sampson, 2008).

5. Premaxilla, height/length ratio ventral to external naris: 0.5–2.0 (0); >2.0 (1) (Carrano & Sampson, 2008).

6. Maxilla, proportions/presence of the anterior ramus: absent (0); anteroposteriorly long (1); or tall and blunt (2) (Carrano & Sampson, 2008).

7. Maxilla, ascending process: posterodorsally directed, with strongly convex anterior and concave posterior margin (0); or subvertical, with almost straight anterior and posterior margins (1) (modified from Bonaparte and Novas 1985).

8. Maxilla, extension of ascending process: large, reaching posteriorly to more than half the length of the maxilla (0); reduced, posterodorsal tip ends well before the half-length of the maxilla (1) (modified from Canale et al., 2009).

9. Maxilla, facet for nasal articulation: shallow, anterolateral (0); socket, lateral (1) (Carrano & Sampson, 2008).

10. Maxilla, palatal process: long and ridged (0); reduced, small, and blunt (1) (modified from Carrano & Sampson, 2008).

11. Maxilla, anteroventral border of antorbital fenestra: graded or stepped (0); demarcated by raised ridge (1) (Carrano & Sampson, 2008).

12. Maxilla, antorbital fossa: extends to the anterior margin of the ascending process, at least in its dorsal part (0); separated from the anterior margin of the ascending process (1) (modified from Canale et al., 2009).

13. Maxilla, ventral portion of antorbital fossa: present on maxilla (0); strongly reduced or absent (1) (modified from Carrano & Sampson, 2008).

14. Anteroposterior length of maxillary–jugal contact, relative to total maxilla length: less tan 40% (0); more than 40% (1) (Carrano & Sampson, 2008).

15. Inclination of maxilla–jugal contact: low, less than 45° from the horizontal (0); steep, more tan 45° from the horizontal (1) (Canale et al., 2009).

16. Nasal–nasal contact in adults: separate (0); partly or fully fused (1) (Carrano & Sampson, 2008).

17. Nasal, row of foramina on dorsal surface: absent (0); present (1) (Carrano & Sampson, 2008).

18. Nasal, posterior narial margin: fossa (0); laterally splayed hood (1) (Carrano & Sampson, 2008).

19. Location of nasal–frontal contact relative to highest point of orbit: anterior (0); directly above (1) (Carrano & Sampson, 2008).

20. Prefrontal, condition in adults: separate (0); partly or completely fused (1) (Carrano & Sampson, 2008).

21. Frontals: unfused (0); or fused (1) (Canale et al., 2009).

22. Frontal–parietal contact in adults: separate (0); fused at least on the dorsal surface (1) (modified from Carrano & Sampson, 2008).

23. Skull roof dorsoventral thickness: thin, relatively flat (0); thickened (1) (Carrano & Sampson, 2008).

24. Skull roof ornamentation: none (0); midline (1); lateral (2) (Carrano & Sampson, 2008).

25. Arrangement of bones along dorsal margin of orbit: postorbital and lacrimal separated by frontal, which forms part of orbital rim (0); contact between postorbital and lacrimal that excludes frontal from orbital rim (1) (Carrano & Sampson, 2008).

26. Small fenestra in the skull roof placed between prefrontal, frontal, postorbital, and lacrimal: absent (0); or present (1) (Sereno et al., 2004; Canale et al., 2009).

27. Supratemporal fossa, bulge on the anteromedial rim: absent, mainly straight border or slightly developed (0), or present forming a pronounced protuberance (1) (modified by Gianechini et al., 2021).

28. Knob-like dorsal projection of parietals and supraoccipital: absent (0); or present (1) (Carrano & Sampson, 2008).

29. Parietal, median separation of the supratemporal fenestrae: flat, broad and homogeneous along all its length without narrow sagittal crest (0), or anteriorly widening subtriangular plate and without posterior narrow sagittal crest (1), or anteriorly widening triangular plate and with a low posterior narrow sagittal crest (2). (ordered) (modified by Filippi et al., 2016).

30. Parietal, size and elevation of nuchal wedge and parietal alae: moderate (0); or tall and slightly extended above frontoparietal skull table (1); or tall and considerably extended above fronto-parietal skull table (2) (ordered) (Filippi et al., 2016).

31. Size of infratemporal fenestra: subequal to size of orbit or smaller (0); considerably enlarged, larger than orbit and especially expanded ventrally (1) (modified from Rauhut, 2003).

32. Postorbital, suborbital flange: absent (0); present (1) (Carrano & Sampson, 2008).

33. Postorbital, anteroposterior length relative to height: markedly less (0); equal to or greater (1) (Carrano & Sampson, 2008).

34. Postorbital, length of the posterior process: less than half of the length of the anterior process (0); more than half, but less than the length of the anterior process (1); longer than the anterior process (2) (ordered) (modified from Canale et al., 2009).

35. Postorbital, orientation of posterior edge: vertical (0); strongly sloped anteroventrally at an angle of 25° or more (1) (modified from Carrano & Sampson, 2008).

36. Postorbital, jugal process: with distinct kink in posterior margin at the beginning of the jugal facet (0); jugal facet continuous with posterior margin of the dorsal part of the postorbital (1) (Pol & Rauhut, 2012).

37. Postorbital, anterior margin of ventral process: straight or slightly concave (0); strongly concave (1) (modified from Canale et al., 2009).

38. Postorbital, posterior margin of the ventral (jugal) process: convex in at least its dorsal part (0); straight or slightly concave over its entire length (1) (modified from Canale et al., 2009).

39. Postorbital, strongly inflated and bulked dorsal margin: absent (0); or present (1). (Canale et al., 2009).

40. Postorbital, lateral depression on the posterodorsal part of the junction of the postorbital processes: present (0); absent (1) (Pol & Rauhut, 2012).

41. Postorbital, morphology of anteroventral portion of ventral process: confluent with remainder of process (0); step and fossa present (1) (Carrano & Sampson, 2008).

42. Morphology of dorsalmost postorbital–squamosal contact: smooth (0); knob (1) (Carrano & Sampson, 2008).

43. Angle between dorsal margin of upper temporal arc (formed by the posterior process of postorbital and anterior process of squamosal) and dorsal skull roof: less than 45° (0); or more tan 45° (1) (modified from Canale et al., 2009).

44. Appearance of postorbital–squamosal contact in lateral view: contact edges visible (0); edges covered by dermal expansions (1) (Carrano & Sampson, 2008).

45. Lacrimal, anterior process: includes antorbital fossa and rim (0); antorbital fossa only (1) (Carrano & Sampson, 2008).

46. Lacrimal, lateral fossa: exposed laterally (0); covered by dermal ossifications (1) (Carrano & Sampson, 2008).

47. Lacrimal, suborbital process: absent (0); present (1) (Carrano & Sampson, 2008).

48. Lacrimal, morphology along dorsal orbit rim: flat (0); raised brow or shelf (1) (Carrano & Sampson, 2008).

49. Lacrimal, constriction of ventral ramus: present (0); absent (1) (modified from Canale et al., 2009).

50. Lacrimal, posterior expansion of the ventral part: present, ventral part of posterior margin of lacrimal concave (0); strongly reduced or absent, ventral part of posterior margin straight or slightly convex (1) (modified from Canale et al., 2009).

51. Morphology of jugal–maxilla contact: slot or groove (0); lateral shelf (1) (Carrano & Sampson, 2008).

52. Morphology of jugal–lacrimal articulation: simple butt joint (0); overlapping and pocketed (1) (Carrano & Sampson, 2008).

53. Jugal, minimal height below the orbit: one-third or less of the height of the orbit (0); half the height of the orbit or more (1) (modified from Canale et al., 2009).

54. Jugal, ventral margin of the posterior half: straight or only slightly convex (0); strongly convex (1) (modified from Canale et al., 2009).

55. Jugal, relative lengths of the posterior prongs: upper prong much shorter than lower prong (0); both prongs subequal in length (1) (Carrano & Sampson, 2008).

56. Squamosal, contribution to nuchal crest: absent or minimal (0); present and broad (1) (Carrano & Sampson, 2008).

57. Squamosal, quadrate flange: wraps around quadrate head (0); ends posterior to quadrate head (1) (Carrano & Sampson, 2008).

58. Quadratojugal, dorsoventral proportions of the prongs for jugal: narrow (0); deep (1) (Carrano & Sampson, 2008).

59. Quadratojugal, overlap onto quadrate posteriorly: absent (0); present (1) (Carrano & Sampson, 2008).

60. Quadrate foramen: present (0); absent (1) (Carrano & Sampson, 2008).

61. Ossification of interorbital region: weak or absent (0); extensive (1) (Carrano & Sampson, 2008).

62. Morphology of trigeminal foramen: single (0); partly or fully split (1) (Carrano & Sampson, 2008).

63. Vagal canal opening: through otoccipital (0); onto occiput (1) (Carrano & Sampson, 2008).

64. Basisphenoid, depth of basisphenoid recess: shallow (0); deep (1) (Carrano & Sampson, 2008).

65. Basisphenoid, shape of opening for basisphenoid recess: ovoid (0); teardrop-shaped (1) (Carrano & Sampson, 2008).

66. Depth of indentation between basal tubera and basisphenoid processes: deep notch (0); shallow embayment (1) (Carrano & Sampson, 2008).

67. Medial fossa ventral to occipital condyle: absent (0); present (1) (Carrano & Sampson, 2008).

68. Size of dorsal groove on occipital condyle: narrow (0); wide (1) (modified by Carrano & Sampson, 2008).

69. Orientation of basioccipital–basisphenoid suture: oblique (0); horizontal (1) (Carrano & Sampson, 2008).

70. Supraoccipital, depth of median ridge: less than width (0); greater than width (1) (Carrano & Sampson, 2008).

71. Palatine, morphology of jugal process: tapered process, triradiate palate (0); expanded process, tetraradiate palatine (1) (Carrano & Sampson, 2008).

72. Pterygoid, pocket on ectopterygoid flange: absent (0); present (1) (Carrano & Sampson, 2008).

73. Shape of pterygoid articulation with basipterygoid process: tab-like (0); acuminate (1) (Carrano & Sampson, 2008).

74. Ectopterygoid, arrangement of jugal and pterygoid processes: oblique (0); parallel (1) (Carrano & Sampson, 2008).

75. Ectopterygoid, proportions: gracile (0); robust (1) (Carrano & Sampson, 2008).

76. Ectopterygoid, ventral excavation: absent (0); fossa (1); groove (2) (Carrano & Sampson, 2008).

77. Size of external mandibular fenestra: small to moderate (0); large (1) (Carrano & Sampson, 2008).

78. Position of anterior end of external mandibular fenestra relative to last dentary tooth: posterior (0); ventral (1) (Carrano & Sampson, 2008).

79. Surangular, horizontal ridge on lateral surface, below mandibular joint: weak or moderate (0); strong (1) (Carrano & Sampson, 2008).

80. Splenial, contour of posterior edge: straight (0); curved or notched (1) (Carrano & Sampson, 2008).

81. Splenial, prongs at anterior end: one (0); two (1) (Carrano & Sampson, 2008).

82. Morphology of dentary–surangular articulation just above external mandibular fenestra: small notch (0); large socket (1) (Carrano & Sampson, 2008).

83. Dentary, shape of articulated rami in dorsal view: V–shaped (0); U–shaped (1) (Carrano & Sampson, 2008).

84. Dentary, ventral margin: straight or only slightly convex (0); strongly convex anteroposteriorly (1) (Canale et al., 2009).

85. Dentary, position of lateral groove: at or above mid-depth (0); in ventral half (1) (Carrano & Sampson, 2008).

86. Dentary, position of posterior end of posteroventral process relative to posterior end of posterodorsal process: far posterior (0); directly ventral (1) (Carrano & Sampson, 2008).

87. Arrangement of premaxillary tooth carinae: nearly symmetrical, on opposite sides (0); more asymmetrical, both on lingual side (1) (Carrano & Sampson, 2008).

88. Number of maxillary teeth: more than 12 (0); 12 or fewer (1) (Carrano & Sampson, 2008).

89. Tooth curvature: substantially curved mesial and distal profiles, with apex positioned distal to distal profile (0); or apex centrally positioned, with mesial profile exhibiting strong curvature and distal profile straight or very slightly curved (1) (modified from Smith et al., 2007; Canale et al., 2009).

90. Surface texture of paradental plates: smooth (0); vertically striated or ridged (1) (Carrano & Sampson, 2008).

91. Visibility of paradental plates in medial view: widely exposed (0); obscured (1) (Carrano & Sampson, 2008).

92. Medial groove in paradental plates exposing replacement teeth: present (0); absent (1) (Carrano & Sampson, 2008).

93. Pronounced size difference between premaxillary and maxillary teeth: absent (0); present (1) (Rauhut, 2004b).

94. Transverse flattening of lateral maxillary teeth: moderate (0); extreme (1) (Rauhut, 2004a,b).

95. Size of largest maxillary tooth: less (0); or more than height of the dentary (1) (Rauhut, 2004a,b).

96. Neural arch pneumaticity: moderate (0); extreme (1) (Carrano & Sampson, 2008).

97. Internal structure of presacral vertebrae: solid (0); camerate (1); camellate (2) (Carrano & Sampson, 2008).

98. Cervical vertebrae, atlantal epipophysis: short and triangular in lateral outline (0); strongly elongate and rod-like (1) (modified from Canale et al., 2009).

99. Cervical vertebrae, length of axial epipophyses: moderate (0); long (1) (Carrano & Sampson, 2008).

100. Cervical vertebrae, morphology of axial spinopostzygapophyseal lamina: weakly concave (0); deeply invaginated (1) (Carrano & Sampson, 2008).

101. Cervical vertebrae, development of axial diapophyses: weak, nubbin (0); prominent, pendant (1) (Carrano & Sampson, 2008).

102. Cervical vertebrae, axial pleurocoels: absent (0); present (1) (Carrano & Sampson, 2008).

103. Cervical vertebrae, postzygodiapophyseal lamina in axis: absent (0); present (1) (Canale et al., 2009).

104. Cervical vertebrae, anterior pleurocoel in postaxial cervical vertebrae: absent (0); fossa (1); foramen (2) (modified from Gauthier, 1986).

105. Posterior pleurocoel in postaxial presacral vertebrae: absent (0); fossa only (1); fossa with pneumatic foramen (2) (Carrano & Sampson, 2008).

106. Cervical vertebrae, anterior articular surface of postaxial cervical vertebrae: concave (0); flat (1); convex (2) (modified from Rauhut, 2003).

107. Cervical vertebrae, shape of postaxial cervical zygapophyses: anteroposteriorly elongated or ovate (0); mediolaterally elongated with lateral half greatly expanded anteroposteriorly (1) (Farke & Sertich, 2013).

108. Cervical vertebrae, demarcation of dorsal surface of neural arch from diapophyseal surface in anterior vertebrae: gently sloping (0); ridge (1) (Carrano & Sampson, 2008).

109. Cervical vertebrae, ratio between anterior width and height of middle and posterior centra: less than 1.3 (0); more than 1.3 (1) (modified from Rauhut, 2003).

110. Cervical vertebrae, anteroposterior position of the neural spines: posterior half of centrum (0); anterior half of centrum (1) (Carrano & Sampson, 2008).

111. Cervical vertebrae, ventral keel on anterior cervicals: present (0); faint or absent (1) (Carrano & Sampson, 2008).

112. Cervical vertebrae, anterior prongs on postaxial epipophyses: absent (0); present (1) (Carrano & Sampson, 2008).

113. Cervical vertebrae, development of pre- and postspinal fossae in postaxial vertebrae: narrow (0); broad (1) (Carrano & Sampson, 2008).

114. Cervical vertebrae, postzygapophysis in mid-cervical vertebrae: placed at least partially over the posterior end of the neural arch pedicle and does not overhang the centrum posteriorly (0); posterior to the neural arch pedicle and overhangs the centrum posteriorly (1). (modified from Carrano et al., 2002; Canale et al., 2009).

115. Cervical vertebrae, position of zygapophyses: close to midline (0); placed far laterally (1) (Carrano & Sampson, 2008).

116. Cervical vertebrae, morphology of anterior epipophyses: low, blunt (0); long, thin (1); long, robust (2) (ordered) (Carrano & Sampson, 2008).

117. Cervical vertebrae, height of mid-cervical epipophyses: low, less than one-third of the height of the neural arch (as measured from the dorsal margin of the centrum to the lateral edge of the postzygapophysis) (0); high, more than half the height of the neural arch (1). (Rauhut & Carrano, 2016).

118. Cervical vertebrae, length/height ratio of mid-centra: less than 2 (0); 2–3 (1); more than 3 (2) (modified from Carrano & Sampson, 2008) (ordered).

119. Cervical vertebrae, height of postaxial neural spines: moderate or tall (0); short (1) (Carrano & Sampson, 2008).

120. Cervical vertebrae, postzygodiapophyseal lamina in mid-vertebrae: present, and connects the diapophysis with the postzygapophysis (0); reduced, indicated by a low ridge in parts (1) (modified from Canale et al., 2009: character 57).

121. Cervical vertebrae, accessory fossa on dorsal surface of postaxial transverse processes: present (0); absent (1) (Carrano & Sampson, 2008).

122. Cervical vertebrae, constriction of posterior vertebrae: moderate, centrum width at mid-length half the width at posterior end or more (0); extreme, mid-centrum width one-third or less (1) (Rauhut & Carrano, 2016).

123. Dorsal vertebrae, anterior pneumatic recess in anterior dorsals: absent (0); present (1) (modified from Rauhut, 2003).

124. Dorsal vertebrae, shape of anterior articular surface of centrum in vertebrae D1 and D2: subcircular (0); dorsoventrally compressed, width/height ratio 1.15 or more (1) (modified from Farke & Sertich, 2013).

125. Dorsal vertebrae, anteroposterior length of articular facet of prezygapophysis in posterior dorsal vertebrae: anteroposteriorly longer than mediolaterally wide (0); anteroposterior length equal to or less than mediolateral width (1) (Baiano et al., 2020).

126. Dorsal vertebrae, prezygapophysis with a ventral process: absent (0); present (1) (Baiano et al., 2020).

127. Dorsal vertebrae, shape of the transverse processes in dorsal view: rectangular (0); triangular (1) (Carrano & Sampson, 2008).

128. Dorsal vertebrae, height of the parapophyses: slightly elevated from centrum (0); project far laterally (1) (Carrano & Sampson, 2008).

129. Dorsal vertebrae, paradiapophyseal lamina: absent, weak (0); pronounced (1) (Carrano & Sampson, 2008).

130. Dorsal vertebrae, centrum length relative to height: more than 1.5 (0); less than 1.5 (1) (modified from Rauhut, 2003).

131. Dorsal vertebrae, infradiapophyseal fossa of middle and posterior vertebrae: undivided (0); divided by paradiapophyseal lamina (1) (Farke & Sertich, 2013).

132. Dorsal vertebrae, orientation of prezygapophyses in posterior half of the series in anterior view: dorsal or dorsomedial (0); dorsolateral (1) (Farke & Sertich, 2013).

133. Sacral vertebrae, number: five or less (0); six or more (1) (modified from Carrano & Sampson, 2008).

134. Sacral vertebrae, transverse dimensions of mid-centra relative to other sacrals: equivalent (0); constricted (1) (Carrano & Sampson, 2008).

135. Sacral vertebrae, orientation of ventral margin of mid-centra: horizontal (0); arched (1) (Carrano & Sampson, 2008).

136. Sacral vertebrae, dorsal edge of neural spines: as thin as remainder of spine (0); thickened (1) (Carrano & Sampson, 2008).

137. Sacral vertebrae, condition of neural spines in adults: separate (0); fused (1) (Carrano & Sampson, 2008).

138. Sacral vertebrae, pneumaticity of neural spines: weak or absent (0); well developed (1) (Carrano & Sampson, 2008).

139. Caudal vertebrae, neural spines of anterior caudal vertebrae in comparison to last dorsal vertebrae: approximately of equal anteroposterior length (0); two thirds or less the length of last dorsal neural spines (1) (modified from Carrano & Sampson, 2008).

140. Caudal vertebrae, longitudinal ventral groove in anterior caudal vertebrae: present (0); absent (1) (modified from Rauhut, 2003).

141. Caudal vertebrae, centrodiapophyseal laminae in anterior mid-caudal vertebrae: absent (0); only anterior centrodiapophyseal lamina present (1); anterior and posterior centrodiapophyseal laminae present as low, rounded ridges (2). (ordered) (modified from Rauhut et al., 2003).

142. Caudal vertebrae, proportions of anterior caudal neural arch base relative to mid-centrum proportions: smaller (0); equal or greater (1) (modified from Carrano & Sampson, 2008).

143. Caudal vertebrae, hyposphene–hypantrum articulations in anterior to mid-caudal vertebrae: absent (0); present (1) (Canale et al., 2009: character 80).

144. Caudal vertebrae, distal morphology of anterior to mid-caudal transverse processes: tapering (0); expanded posteriorly (1) (modified from Carrano & Sampson, 2008).

145. Caudal vertebrae, anterior process at distal end of anterior to mid-caudal transverse processes: absent (0); present (1) (modified from Carrano & Sampson, 2008).

146. Caudal vertebrae, neural spines of mid-caudals: inclined posteriorly (0); low and rectangular (1); anteroposteriorly short and vertical (2) (modified from Rauhut, 2003).

147. Caudal vertebrae, proximodistal length of anterior transverse processes: less than 1.4 times the length of caudal centra (0); more than 1.4 times the length of caudal centra (1) (Rauhut et al., 2003).

148. Ribs, contact between cervical vertebrae and cervical ribs in adults: separate (0); fused (1) (Carrano & Sampson, 2008).

149. Ribs, wing-like process at the base of the anterior cervical rib shafts: absent (0); present (1) (Carrano & Sampson, 2008).

150. Ribs, bifurcate cervical rib shafts: absent (0); present (1) (Carrano & Sampson, 2008).

151. Scapula, shaft length/width ratio: wide shaft, length of shaft five times or less its width (0); narrow shaft, length of the shaft more than five times its own width (1) (Aranciaga Rolando et al., 2021).

152. Scapula, large vertical depression on lateral side above the glenoid: absent (0); present (1) (Pol & Rauhut, 2012).

153. Scapula, distal expansion: present (0); absent (1) (Gauthier, 1986).

154. Scapula, relative width of the blade: broad, more than twice glenoid depth (0); narrow, less than twice glenoid depth (1) (Carrano & Sampson, 2008).

155. Scapulocoracoid, glenoid orientation: ventral, main axis of glenoid parallel or almost parallel to the scapulocoracoid shaft (0); posteroventral, main axis of glenoid forming an acute angle respect to scapulocoracoid shaft (1) (modified by Aranciaga Rolando et al., 2021).

156. Coracoid, development of posteroventral process: moderate (0); pronounced (1) (Carrano & Sampson, 2008).

157. Coracoid, spacing between glenoid lip and posteroventral process: close, less than half of the length of the glenoid (0); wide, more than half of the length of the glenoid (1) (modified from Carrano & Sampson, 2008).

158. Coracoid, ratio between height (as measured over the glenoid) and length: less than 1.8 (0); more than 1.8 (1) (modified from Carrano & Sampson, 2008).

159. Humerus, shape of head in proximal view: transversely elongate, more than two times wider than long anteroposteriorly (0); stout, less than two times wider than long (1); globular (2) (ordered) (modified from Carrano & Sampson, 2008).

160. Humerus, shape of distal condyles: rounded (0); flattened (1) (Carrano & Sampson, 2008).

161. Humerus, placement of greater tubercle: proximally, usually confluent with proximal articular surface (0); offset distally, separated from articular surface further than the medial tuberosity (1) (modified from Carrano & Sampson, 2008).

162. Humerus, posterolateral tubercle on the proximal part: absent or poorly developed as a low mound (0); well-developed, sharply defined tubercle (1) (Novas et al., 2006).

163. Humerus, shape in lateral view: S–shaped (0); straight (1) (Rauhut, 2003).

164. Humerus, shape in anterior view: with a concave or straight lateral margin and a mildly concave medial margin (0); with a convex lateral and strongly concave medial margin (1) (Rauhut & Carrano, 2016).

165. Humerus, longitudinal torsion of the shaft: absent (0); present (1) (Carrano & Sampson, 2008).

166. Humerus, size of deltopectoral crest: prominent flange (0); reduced (1) (Carrano & Sampson, 2008).

167. Humerus, length relative to femur length: more than one-third (0); less than one-third (1) (Carrano & Sampson, 2008).

168. Radius, length: more than half the length of the humerus (0); equal to or less than half the length of the humerus (1) (Rauhut, 2003).

169. Radius, distal articular surface: flat or slightly concave (0); strongly convex (1) (modified from Canale et al., 2009: character 90).

170. Ratio between maximal length of metacarpus and length of humerus: more than 23% (0); 15–23% (1); less than 15% (2) (ordered) (modified from Pol & Rauhut, 2012).

171. Metacarpal I, distal end: strongly asymmetrical, lateral condyle considerably higher dorsoventrally and more distally expanded than medial condlye (0); asymmetry reduced, lateral and medial condyles of subequal size or indistinct (1). (Rauhut & Carrano, 2016).

172. Metacarpal II, ventromedial expansion of medial distal condyle: present (0); absent (1) (Pol & Rauhut, 2012).

173. Metacarpal II and metacarpal III, pronounced rim on proximal border of extensor fossa: absent (0); present (1) (Rauhut & Carrano, 2016).

174. Metacarpal III, distal articular end in dorsal view: broad, with two condyles that are more or less symmetrical (0); broad, lateral condyle extends further distally than medial condyle (1); reduced in width, single convex articular surface (2). (ordered) (Rauhut & Carrano, 2016).

175. Metacarpal IV: slender, width of mid-shaft less than half of the width of mid-shaft of metacarpal II (0); robust, width more than half of that of metacarpal II (1) (Pol & Rauhut, 2012).

176. Manual phalanx II–1, relative length: more than twice width (0); less than twice width (1) (modified from Carrano & Sampson, 2008).

177. Constricted neck between articular ends of manual phalanges in digits II and III: present (0); absent, phalanges very stout and broad (1) (modified from Pol & Rauhut, 2012).

178. Gynglimoidal distal articular surface of proximal manual phalanges: well developed, condyles separated by deep notch (0); reduced, notch weak or absent (1) (modified from Canale et al., 2009: character 93).

179. Contacts between pelvic elements in adults: separate (0); at least partially fused (1) (Carrano & Sampson, 2008).

180. Ratio of length of femur to length of ilium: more than 1.3 (0); less than 1.1 (1) (modified from Canale et al., 2009: character 82).

181. Ilium, posterior width of the brevis fossa: subequal to anterior width (0); twice anterior width (1) (Carrano & Sampson, 2008).

182. Ilium, lateral brevis shelf: directed mainly ventrally, postacetabular blade of the ilium faces mainly laterally (0); flares laterally, postacetabular blade of the ilium faces dorsolaterally (1) (Rauhut & Carrano, 2016).

183. Ilium, morphology of lateral surface between supraacetabular crest and brevis shelf: gap (0); continuous (1) (Carrano & Sampson, 2008).

184. Ilium, anterior margin: faces anteriorly or anterodorsally (0); faces anteroventrally (1) (Pol & Rauhut, 2012).

185. Ilium, shape of the posterior margin: convex (0) or straight to slightly concave or undulating (1), or deeply notched (2) (ordered) (modified by Carrano & Sampson, 2008; Tortosa et al., 2014).

186. Ilium, shape of dorsal margin of central iliac blade: convex (0); straight (1) (modified from Carrano & Sampson, 2008).

187. Relative sizes of iliac–pubic and iliac–ischial articulations: subequal (0); iliac–pubic articulation anteroposteriorly larger (1) (Carrano & Sampson, 2008).

188. Relative proximodistal length of pubic and ischial peduncle: pubic peduncle considerably longer than ischial peduncle (0); peduncle of subequal length, or ischial peduncle longer (1) (Pol & Rauhut, 2012).

189. Ilium, orientation of ischial peduncle: posteroventrally inclined (0); vertical (1) (Pol & Rauhut, 2012).

190. Ilium, shape of anterior margin of iliac preacetabular process: rounded (0); undulating (1) (Carrano & Sampson, 2008).

191. Ilium, anteroventral lobe of iliac preacetabular process: absent (0); present (1) (Carrano & Sampson, 2008).

192. Ilium, orientation of the ventral edge of the lateral brevis shelf: oriented strongly posteroventrally (0); either horizontally or slightly posteroventrally directed (1); or anteroventrally directed (2) (ordered) (modified by Tortosa et al., 2014).

193. Pubis, contact between pubic apices: separate (0); contacting (1) (Carrano & Sampson, 2008).

194. Morphology of contact between pubis and ilium: planar (0); peg-and-socket (1) (Carrano & Sampson, 2008).

195. Pubis, morphology of dorsal surface of pubic boot on midline: convex (0); concave (1) (Carrano & Sampson, 2008).

196. Ischium, proximal end: pubic articular facet dorsoventrally deeper than that for the ilium (0); iliac articular facet dorsoventrally deeper than that for the pubis (1) (Langer et al., 2019).

197. Ischium, notch ventral to obturator process: absent (0); present (1) (Carrano & Sampson, 2008).

198. Ischium, morphology of distal end: rounded, separate (0); expanded, with triangular boot (1) (modified from Carrano & Sampson, 2008).

199. Morphology of contact between ischium and ilium: planar (0); peg-and-socket (1) (Carrano & Sampson, 2008).

200. Proportions of limb bones: moderate to gracile (0); robust (1) (Carrano & Sampson, 2008).

201. Dimorphism in hindlimb morphology: absent (0); present (1) (Carrano & Sampson, 2008).

202. Femur, fourth trochanter: developed as a high flange or crest (0); reduced to a low ridge (1) (Bonaparte, 1991a,b; Canale et al., 2009: character 96).

203. Femur, orientation of fourth trochanter: posterior (0); posteromedial (1) (Ibiricu et al., 2021).

204. Femur, morphology of anterolateral muscle attachments on proximal end: continuous trochanteric shelf (0); distinct lesser trochanter and attachment bulge (1) (Carrano & Sampson, 2008).

205. Femur, development of medial epicondyle: rounded (0); ridge (1); long flange (2) (ordered) (Carrano & Sampson, 2008).

206. Femur, morphology and orientation of the tibiofibularis crest: narrow, longitudinal (0); broad, oblique (1) (Carrano & Sampson, 2008).

207. Femur, popliteal fossa in adults: smooth (0), or traversed by infrapopliteal ridge between medial (tibial) distal condyle and tibiofibular crest (1) (Tortosa et al., 2014).

208. Tibia, cnemial crest: distal end reduced proximodistally (0); distal end expanded proximodistally, cnemial crest almost rectangular in shape (1); distal end expanded proximodistally with a ventral process, cnemial crest has an axe-like outline (2) (ordered) (modified by Carrano & Sampson, 2008; Rauhut & Carrano, 2016).

209. Tibia, expansion of cnemial crest (as measured from the level of the tibial shaft): ratio tibial length/cnemial crest >6.5 (0); ratio tibial length/cnemial crest <6.5 (1) (modified from Canale et al., 2009: character 99).

210. Tibia, proximodistally directed ridge on the medial surface of cnemial crest: absent (0); present (1) (Cerroni et al., 2022; Baiano et al., 2022).

211. Tibia, shape of the distal end in distal view: rounded (0); mediolaterally elongate (1) (Carrano & Sampson, 2008).

212. Tibia, distal end in anterior view: with a marked step running obliquely at an angle of 30 degrees or more (0); step originating more proximally and oriented nearly mediolaterally (1); anterior surface of distal tibia flat (2) (Ibiricu et al., 2021).

213. Fibula, development of fibular fossa on medial aspect of proximal end: posterior groove (0); posteriorly open fossa (1); medially open fossa (2) (Carrano & Sampson, 2008).

214. Fibula, size of iliofibularis tubercle: moderate (0); large (1) (Carrano & Sampson, 2008).

215. Contact between fibula and ascending process of astragalus in adults: separate (0); fused (1) (Carrano & Sampson, 2008).

216. Astragalus, morphology of ascending process: blocky (0); laminar (1) (Carrano & Sampson, 2008).

217. Astragalus, orientation of distal condyles: ventral (0); anteroventral (1) (modified from Carrano & Sampson, 2008).

218. Astragalus, horizontal groove across the anterior face of the condyles: absent or weak (0); pronounced (1) (Carrano & Sampson, 2008).

219. Contact between astragalus and calcaneum in adults: separate (0); fused (1) (Carrano & Sampson, 2008).

220. Astragalus, development of articular surface for distal end of fibula: large, dorsal (0); reduced, lateral (1) (Carrano & Sampson, 2008).

221. Astragalus, height of the ascending process relative to depth of astragalar body: less or equal (0); greater (1) (Carrano & Sampson, 2008).

222. Distal tarsal III, partially or totally co-ossified with the metatarsal III in adults: absent (0); present (1) (Cerroni et al., 2022; Baiano et al., 2022).

223. Metatarsal II, width of shaft relative to width of metatarsal III: subequal (0); reduced (1) strongly reduced (2) (ordered) (modified by Carrano & Sampson, 2008).

224. Metatarsal II, proximal articular surface: subequal in width to that of metatarsals III and IV (0); two-thirds or less the width of metatarsals III or IV (1) (modified from Carrano & Sampson, 2008: character 148).

225. Metatarsal III, outline of the distal articular surface in distal view: subquadrangular, or slightly wider than tall (0); rectangular, much wider than tall (1) (Cerroni et al., 2022; Baiano et al., 2022).

226. Metatarsal II, III, and IV, rugose scar for the *Mm. gastrocnemii pars medialis et lateralis* in the distal part of posterior surface: slightly rugose (0); prominent, forming a distinct step on both metatarsal (1) (Cerroni et al., 2022; Baiano et al., 2022).

227. Metatarsal IV, shaft: uncompressed (0), lateromedially compressed with respect to the shaft of metatarsal III (1) (Langer et al., 2019).

228. Metatarsal IV, distal end: strongly asymmetrical, medial condyle much broader and more distally expanded than lateral condyle (0); more symmetrical, condyles subequal (1) (modified from Canale et al., 2009: character 112).

229. Metatarsal IV, width of distal end: subequal (0), or less than 50% width of distal condyles of metatarsal II and III (1) (Tortosa et al., 2014).

230. Morphology of lateral and medial grooves on pedal unguals: single (0); double (1) (Carrano & Sampson, 2008).

231. Mediolateral symmetry of pedal digit II ungual: symmetrical (0); asymmetrical (1) (Carrano & Sampson, 2008).

232. Ventral surface of pedal unguals; with a well-developed flexor tubercle, and usually flat (0); or without flexor tubercle and with a ventral depression in its proximal end (1) (Novas, Dalla Vecchia & Pais, 2005; Canale et al., 2009: character 113).

233. Length of pedal digit phalanges I–1 + I–2 relative to III–1: greater (0); less than or equal (1) (Carrano & Sampson, 2008).

234. Phalanx IV-1, proximal articular surface: high as wide or slightly higher (0); strongly higher than wide, with a ratio height/width of 1,25 or more (1) (Baiano et al., 2021).

235. Phalanx IV-1, morphology and orientation of hiperextensor pit on the dorsal surface: centrally positioned, being circular or mediolaterally oval in contour (0); laterally positioned, oval in contour with the mayor axis obliquely oriented (1). (Baiano et al., 2021).

236. Odontoid, anterior projection in lateral view: reduced, barely reaches the ventral edge of the axial intercentrum or slightly exceeds it (0); developed, far exceeds it (1) (new).

237. Dorsal vertebrae, orientation of the centroprezygapophyseal lamina in D1-D3 in lateral view: slightly anteriorly inclined, the prezygapophyses are located in correspondence or slightly anteriorly to the anterior rim of the centrum (0); strongly anteriorly inclined, the prezygapophyses exceed considerably the centrum (1) (new).

238. Dorsal vertebrae, D1-D3 in dorsal view: anterior margin of the prezygapophysis continuous with the prezygodiapophyseal laminae (0); discontinuous, with a notch among the prezygapophyses and the prezygodipophyseal lamina (1) (new).

239. Dorsal vertebrae, middle and posterior vertebrae, dorsal end of neural spine in lateral view: rectangular (0); dorsal end expanded anteroposteriorly with a faint anterior and/or posterior interspinous process (1); presence of anterior and posterior well-developed interspinous processes, articulating each other between vertebrae (2) (new).

240. Caudal vertebrae, dorsoventral development of the prezygapophyses-hypantrum complex with respect to the anterior articular surface in anterior vertebrae: reduced, dorsoventral hight less than half of the centrum (0); tall, dorsoventral height more than the half of the centrum (1) (new).

241. Caudal vertebrae, orientation of the prezygapophyses in the first nine caudal vertebrae with respect to the vertical plane in anterior view: inclination less than 50° (0); inclination greater than 50° (1) (new).

242. Caudal vertebrae, scar in the posterlateral end of the dorsal surface in anterior and middle transverse processes: absent (0); present (1) (new).

243. Dorsal vertebrae, middle and posterior vertebrae, height of the neural spine with respect to the centrum: low, height equal or lesser than the height of the centrum (0); tall, higher than the centrum (1) (modified from the character 257 in Filippi et al., 2016, and references therein).

244. Caudal vertebrae, orientation of the transverse processes in anterior portion of the tail: inclined ventrally or laterally (0); or slightly dorsolaterally (0 to ∼20◦) (1); either dorsally (between 30◦ and 50◦) (2); or strongly dorsally (more than 50°) (3) (character 282 in Filippi et al., 2016, and references therein).

245. Caudal vertebrae, ventrolateral ridge at lateral end of anterior transverse processes: absent (0); present (1) (character 283 in Filippi et al., 2016, and references therein).

246. Caudal vertebrae, lateral rim of the anterior and middle transverse processes: straight (0); concave (1); strongly convex/sinusoidal (2) (modified from the character 76 in Canale et al., 2009).
